# A Guided Wave Sensor Enabling Simultaneous Wavenumber-Frequency Analysis for Both Lamb and Shear-Horizontal Waves

**DOI:** 10.3390/s17030488

**Published:** 2017-03-01

**Authors:** Baiyang Ren, Hwanjeong Cho, Cliff J. Lissenden

**Affiliations:** Department of Engineering Science and Mechanics, The Pennsylvania State University, University Park, PA 16802, USA; huc146@psu.edu

**Keywords:** Lamb waves, shear horizontal waves, wavenumber-frequency analysis, PVDF sensor

## Abstract

Guided waves in plate-like structures have been widely investigated for structural health monitoring. Lamb waves and shear horizontal (SH) waves, two commonly used types of waves in plates, provide different benefits for the detection of various types of defects and material degradation. However, there are few sensors that can detect both Lamb and SH waves and also resolve their modal content, namely the wavenumber-frequency spectrum. A sensor that can detect both waves is desirable to take full advantage of both types of waves in order to improve sensitivity to different discontinuity geometries. We demonstrate that polyvinylidene difluoride (PVDF) film provides the basis for a multi-element array sensor that detects both Lamb and SH waves and also measures their modal content, i.e., the wavenumber-frequency spectrum.

## 1. Introduction

Ultrasonic guided waves have been widely researched for structural health monitoring (SHM) [1] because of their large volume coverage and good sensitivity to various defects. Plate-like structures are one of the most common load-bearing components, and their integrity is prone to degradation so it needs to be monitored. Guided waves in isotropic traction-free plates are categorized as Lamb waves and shear horizontal (SH) waves. Lamb waves have particle motion in the sagittal plane, while SH wave particle motion is in the transverse in-plane direction [2]. This substantial difference in wave polarization provides them with sensitivities to different types and orientations of defects.

Lamb waves have been studied both theoretically and experimentally for their capability to detect and characterize notches and surface cracks [3,4] oriented perpendicular to the wave vector. On the other hand, SH waves can be employed to detect cracks that are either normal [5] or parallel [6] to the wave vector. For the latter case, the size of the defect can be characterized through the scattered SH wave. We emphasize that when the wave vector for a Lamb wave is parallel to a crack, there is very little interaction with the crack because no particle motion occurs perpendicular to the crack faces. Thus, there is a substantial benefit to employing both Lamb and SH waves for improved defect sensitivity. Additionally, a wave-defect interaction usually results in mode conversion and thus a change in wavelength [3]. The mode conversion details usually correspond to valuable characteristic information of the defects. It is difficult to designate a wavelength to the receiver without the knowledge of defects, which is usually the case in the field. Therefore, to not lose any information about defects, it is desirable to receive all wavelengths and resolve them in post processing. A receiver can detect both Lamb and SH waves and also resolve them in the wavenumber domain is the objective of this work.

Piezoelectric transducers are widely employed for guided wave applications in SHM. Lamb waves can readily be excited using round or rectangular piezoelectric wafers and array transducers [7,8,9,10]. Mode control and direction control can also be realized by using a comb configuration [9,10] or phase-delay excitation [11,12]. However, the transduction of SH waves needs shear-type piezoelectric devices. Kamal and Giurgiutiu [13] employed thickness-shear mode piezoelectric plate to excite SH waves. Zhou et al. [14] used lead magnesium niobate-lead titanate (PMN-PT) single crystals as the actuator and the sensor. The PMN-PT intrinsically has a non-zero $d_{36}$ coefficient and can excite SH0 waves in certain directions. However, the direction for SH0 mode excitation cannot be the same as Lamb wave modes, namely the A0 and S0 modes, due to the anisotropy of the PMN-PT material. Miao and Li [15] modified lead zirconate titanate (PZT) ceramics to enable $d_{36}$ coefficients via ferroelastic domain engineering. Piezoelectric fiber composites are also used to enable the shear mode activation of a multilayer patch [16]. However, it has been noted that due to the finite size of the transducer or the existence of other piezoelectric coupling coefficients, it is challenging to generate a pure SH wave without also exciting Lamb-type waves [13]. It is beneficial and important to differentiate these two types of waves through the receiver and signal processing to reduce confusion and misinterpretation of the signal and further improve the sensitivity to defects.

PVDF interdigital transducers (IDTs) [17,18] and PVDF comb transducers [19] have been applied to curved surfaces. While piezoelectric ceramics are usually brittle and cannot conform to curved surfaces, piezoelectric fiber composites (PFC) [20] with interdigital electrodes can send and receive both Lamb waves [21] and SH waves [16] and can have sufficient flexibility for surfaces with moderate curvature. However, the interdigital or comb electrodes are preferentially tuned to wavelengths equal to the electrode spacing. Thus, these types of transducers may not receive converted modes with a wavelength different than the incident wave. Stepinski et al. [22] showed a tunable IDT where the IDT pitch is variable, enabling it to receive different wavelengths. However, the wavelength range that can be received is somewhat limited. Instead of interdigital electrodes, multi-element array electrodes can also be applied to a PFC to make it an array receiver. However, since the PFC usually has higher quality factor Q due to its high piezoelectric coupling coefficient, its bandwidth is expected to be significantly narrower than PVDF. This will limit its use in different frequency ranges. In addition, the piezoelectric fiber in PFC is a good waveguide, and the wave could propagate along the fiber easily and cause severe cross-talk. PVDF has lower cross-talk because it is nearly mechanically isotropic and its polymer nature is not a good waveguide. For these two reasons, PVDF was selected for the multi-element array.

This work focuses on an array sensor able to detect waves polarized as either Lamb or SH waves and determine their wavelength. A uniaxial polyvinylidene difluoride (PVDF) film is employed and rotated ±45° to enable the coupling between in-plane shear deformation and the electric displacement in the out-of-plane direction. Its array configuration guarantees that wavenumber-frequency analysis by signal post-processing enables different modes at the same frequency to be resolved and separated. Such a low-profile, low-mass, conformable, inexpensive sensor is ideal for SHM and other applications [23,24]. Wave actuation could come from any source, although for SHM many of the same features are desirable.

## 2. Piezoelectric Modeling

Piezoelectric constitutive relations can be expressed in different ways. The strain-charge form is:

(1) $$S=s_{E}T+d^{t}E$$

(2) $$D=dT+\varepsilon_{T}E$$

and its stress-charge form is:

(3) $$T=c_{E}S-e^{t}E$$

(4) $$D=eS+\varepsilon_{S}E$$

where S and T are the strain and stress tensors respectively, D and E are electric displacement and electric field vectors respectively, $s_{E}$ is the compliance tensor under constant electric field, $c_{E}$ is the stiffness tensor under constant electric field, $\varepsilon_{T}$ is the dielectric constant under constant stress, $\varepsilon_{S}$ is the dielectric constant under constant strain, d and e are piezoelectric coupling coefficients, and the superscript *t* means transpose.

The material properties for unidirectional PVDF films vary depending on the manufacturing process but a representative set of properties for PVDF film is adopted from [25,26] and listed in Table 1, where the 3-direction is normal to the plane of the film.

Since the $d_{36}$ coefficient is zero for a wave vector in the 1-direction of the PVDF, an SH wave cannot be detected in this direction. However, by rotating the PVDF film it will have an apparent $d_{36}^{\prime }$ that is not zero.

**Figure 1:**
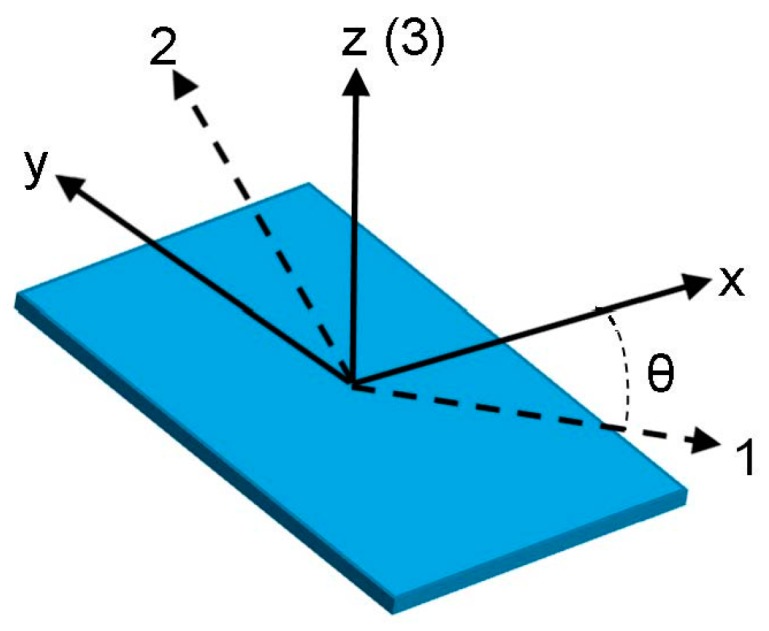
Sketch for the coordinate transformation of PVDF material properties.

To calculate the effective material properties in a rotated coordinate system, the following standard matrix transformations apply. Take the rotation angle to be θ, as shown in Figure 1, and let

(5) c=cos θs=sin θ

For the rotation about the 3-direction, the rotation transformation matrices are [27]:

(6) $$Q=\left[\begin{array}{ccc} c & s & 0\\ -s & c & 0\\ 0 & 0 & 1 \end{array}\right]\;M=\left[\begin{array}{cc} \begin{array}{ccc} c^{2} & s^{2} & 0\\ s^{2} & c^{2} & 0\\ 0 & 0 & 1 \end{array} & \begin{array}{ccc} 0 & 0 & 2cs\\ 0 & 0 & -2cs\\ 0 & 0 & 0 \end{array}\\ \begin{array}{ccc} 0 & 0 & 0\\ 0 & 0 & 0\\ -cs & cs & 0 \end{array} & \begin{array}{ccc} c & -s & 0\\ s & c & 0\\ 0 & 0 & c^{2}-s^{2} \end{array} \end{array}\right]\;N=\left[\begin{array}{cc} \begin{array}{ccc} c^{2} & s^{2} & 0\\ s^{2} & c^{2} & 0\\ 0 & 0 & 1 \end{array} & \begin{array}{ccc} 0 & 0 & cs\\ 0 & 0 & -cs\\ 0 & 0 & 0 \end{array}\\ \begin{array}{ccc} 0 & 0 & 0\\ 0 & 0 & 0\\ -2cs & 2cs & 0 \end{array} & \begin{array}{ccc} c & -s & 0\\ s & c & 0\\ 0 & 0 & c^{2}-s^{2} \end{array} \end{array}\right]$$

where ***Q***, ***M*** and ***N*** are the direction cosines tensor and transformation matrices for the contracted stress and strain vectors respectively.

Then the rotated material property matrices (contracted from the tensors) are [27]:

(7) $$s_{E}^{\prime }=Ns_{E}N^{t}\;c_{E}^{\prime }=Mc_{E}M^{t}\;d^{\prime }=QdN^{t}\;e^{\prime }=QeM^{t}\;\varepsilon_{T}^{\prime }=Q\varepsilon_{T}Q^{t}\;\varepsilon_{S}^{\prime }=Q\varepsilon_{S}Q^{t}$$

**Figure 2:**
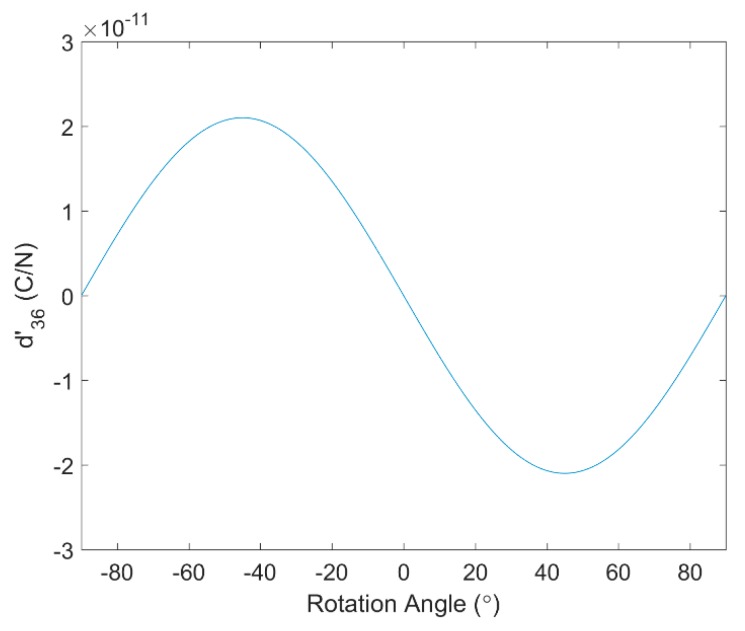
The $d_{36}^{\prime }$ coefficient for the rotation angles from −90° to 90°.

The piezo strain constant $d_{36}^{\prime }$ is plotted in Figure 2 for rotation angles from −90° to 90°. The maximum coupling between in-plane shear stress, which has indices 12 and is contracted to index 6, and the electric displacement in the 3-direction occurs at ±45°. Since +45° and −45° cases have the same magnitude of $d_{36}^{\prime }$, only the +45° case will be considered in the following study. Thus, two PVDF film orientations, 0° and 45°, will be used in the numerical simulation and experiments. The coupling coefficients d and $d^{\prime }$ for 0° and 45° PVDF film are:

(8) $$d=\left[\begin{array}{cc} \begin{array}{ccc} 0 & 0 & 0\\ 0 & 0 & 0\\ 23 & 2 & -33 \end{array} & \begin{array}{ccc} 0 & 0 & 0\\ 0 & 0 & 0\\ 0 & 0 & 0 \end{array} \end{array}\right]\mathrm{pm}/V\;\mathrm{for}\;\theta =0{}^{\circ}\;d^{\prime }=\left[\begin{array}{cc} \begin{array}{ccc} 0 & 0 & 0\\ 0 & 0 & 0\\ 12.5 & 12.5 & -33 \end{array} & \begin{array}{ccc} 0 & 0 & 0\\ 0 & 0 & 0\\ 0 & 0 & -21 \end{array} \end{array}\right]\mathrm{pm}/V\;\mathrm{for}\;\theta =45{}^{\circ}$$

Equation (8) demonstrates that $d_{31,}^{\prime }\;d_{32}^{\prime },\;\mathrm{and}\;d_{36}^{\prime }$ values all depend on θ, while $d_{33,}^{\prime }$ does not. Thus, a comprehensive numerical study on the sensitivity of 0° and 45° PVDF films to both Lamb and SH waves is needed.

## 3. Numerical Simulation

**Figure 3:**
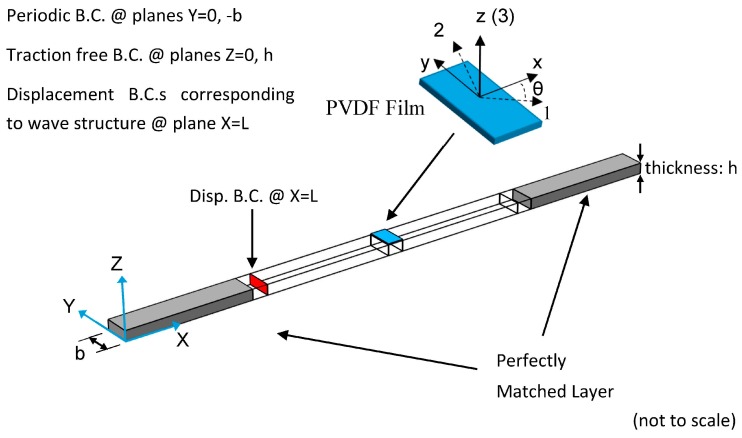
Sketch of the finite element model to predict the response of PVDF film to incident waves.

Multiphysics (elasticity and piezoelectricity) finite element simulations are conducted of PVDF film as a receiver for both Lamb and SH waves propagating in an aluminum plate. In addition to analyzing the effect of film orientation θ, sensitivity to different wavelengths and the effect of PVDF film width are studied. The dimensions of the PVDF film in the x, y and z directions are width, length and thickness respectively, as shown in Figure 3.

### 3.1. Sensitivity to Guided Wave Modes

A frequency domain finite element analysis is performed to evaluate the sensitivity of 0° and 45° oriented PVDF films for receiving Lamb and SH waves based on the voltage magnitude of the received signal from a given incident wave.

The waveguide is a 1mm-thick aluminum plate. The plate is modeled as a 3D strip in the COMSOL Multiphysics^®^ finite element analysis software. The lateral surfaces of the strip have traction free boundary conditions applied to the top and bottom and periodic boundary conditions on the left and right. The advantage of using a strip model is that it enables plane wave propagation of both Lamb and SH waves and it is computationally efficient [24]. Guided wave actuation is through application of displacement boundary conditions to a cross section of the aluminum plate. The applied displacement profile matches the wave structure of the guided wave mode being actuated and the magnitudes are adjusted such that the excited guided wave has a unit power flux throughout the cross section, which ensures that each excited wave mode contains the same amount of energy. Quadratic hexahedron elements with the size of 0.1 mm are used to mesh the aluminum plate. Considering the minimum wavelength existing in the model is 2.3 mm, which corresponds to the A0 mode at 1 MHz. Thus, the wavelength-to-mesh ratio is 23, which is sufficiently high to avoid issues with convergence. The thickness direction of the PVDF film is meshed into two elements to better represent the deformation of the PVDF film. All simulations are conducted in the frequency domain.

To receive the guided wave, a 0.11 mm thick and 1.2 mm wide PVDF film is bonded to the top surface of the aluminum plate. The bottom surface of the PVDF film is the ground and the top surface is assumed to have float potential. Perfectly matched layers (PML) are appended to the two ends of the strip to absorb the wave. Similarly, periodic boundary conditions are also applied to the two lateral surfaces of the PVDF film (with normals in the ±y-direction). The absolute value of the received voltage on the PVDF film is recorded as the response to the unit-power incident wave.

**Figure 4:**
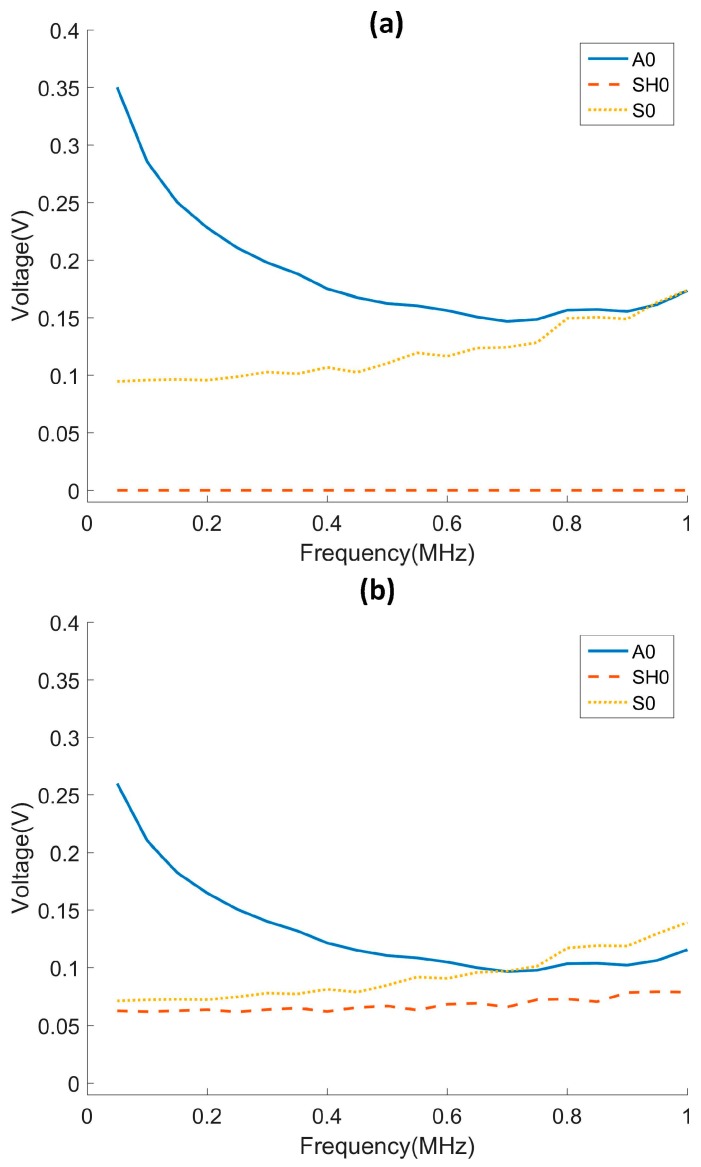
Received voltages with (**a**) 0° and (**b**) 45° PVDF film for Lamb wave modes A0 and S0 and SH mode SH0 for frequencies up to 1 MHz.

**Figure 5:**
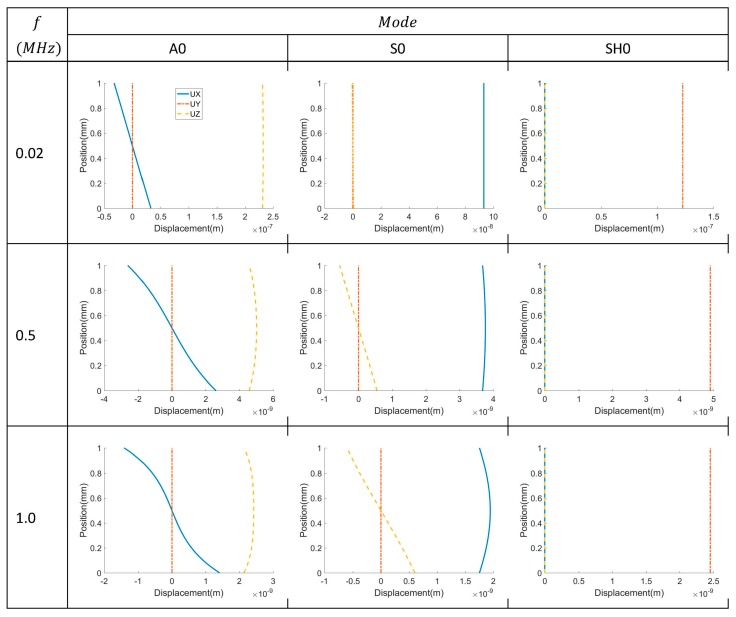
The displacement wave structures of A0, S0 and SH0 modes at 0.02, 0.5 and 1 MHz.

The numerical simulation results for the voltage measured on the top surface of the PVDF for Lamb wave modes A0 and S0 and SH wave mode SH0 are shown in Figure 4 for 0° and 45° oriented PVDF films. The Lamb wave modes are received for both orientations, with the 0° film providing a higher voltage, as expected from Equation (8). However, the SH0 mode can only be detected by the film oriented at 45°, also as expected from Equation (8). Therefore, while PVDF film oriented at 45° has a lower sensitivity to Lamb wave modes, it is more versatile because it is sensitive to both Lamb and SH waves. The displacement wave structures of A0, S0 and SH0 modes at 0.02, 0.5 and 1.0 MHz are shown in Figure 5. The SH0 mode only has the motion polarized in the y-direction while A0 and S0 modes have displacement components in both the x and z-directions.

### 3.2. Width Selection

**Figure 6:**
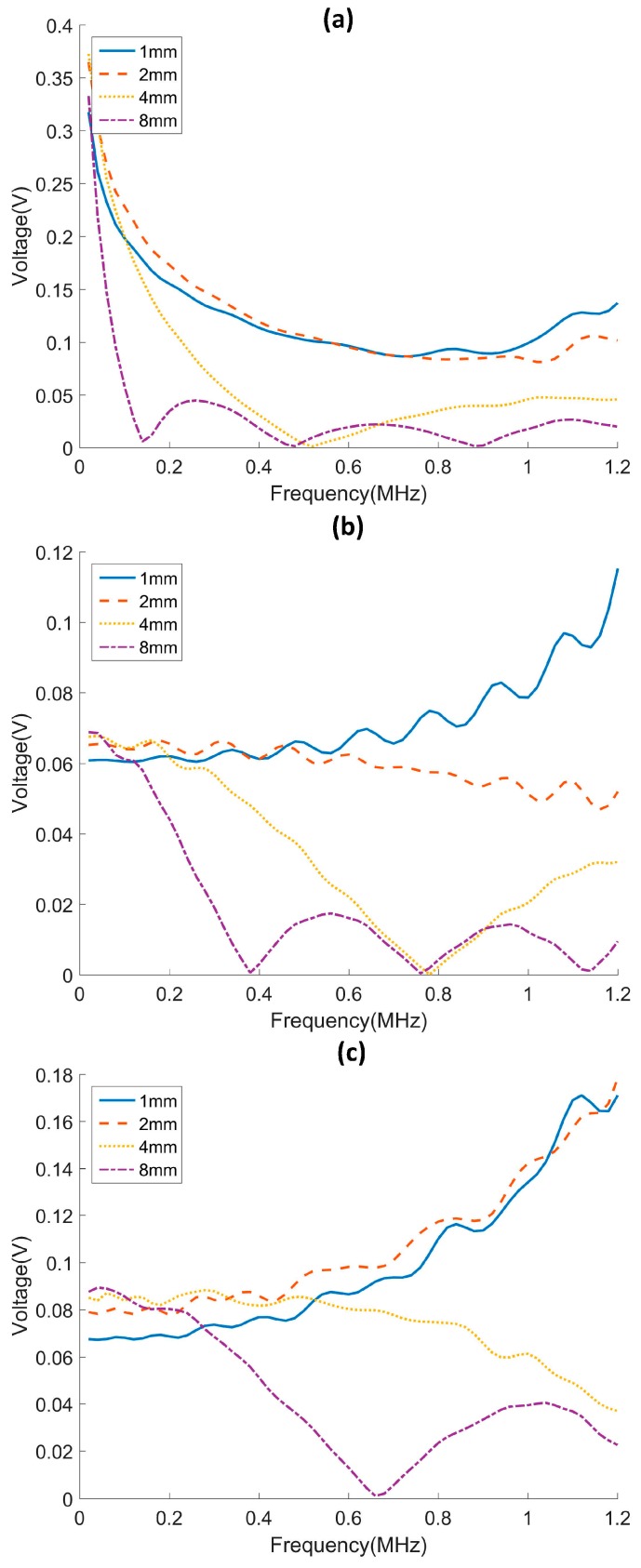
Voltage responses of PVDF films having different widths for (**a**) A0, (**b**) SH0 and (**c**) S0 incidence.

The second numerical study is focused on the selection of an optimized width of PVDF film. The reception of A0, SH0 and S0 modes are analyzed for a 45° PVDF film. The width of the PVDF film is chosen to be 1, 2, 4 and 8 mm. The voltage responses from 0.02 to 1.2 MHz are plotted in Figure 6. It is observed that the response voltage has a minimum whenever the width of the PVDF film is an integer multiple of the wavelength of the incident wave. When the width of an electrode covers an integer multiple of the wavelength, the positive and negative charge generated by the strain will simply cancel out, resulting in zero voltage. Such an effect is evident for the 8 mm cases. On the other hand, the 1 mm and 2 mm cases have relatively uniform sensitivity to different modes over the range of examined frequencies. Thus, it is preferred to use PVDF film with electrodes that have small widths such that a wider bandwidth is obtained.

## 4. Sensor Design and Fabrication

**Figure 7:**
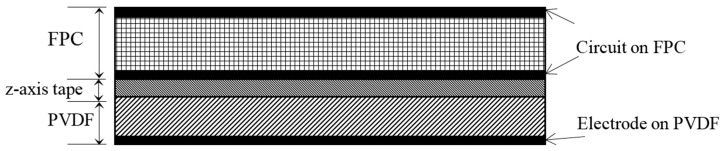
Cross-section of the 3-layer sensor structure.

The Lamb-SH wave sensor has a similar design and assembly to the multi-element Lamb wave sensor used in [23], the only difference being that the PVDF film is oriented at 45° instead of 0°. The three-layer patch, as shown in Figure 7, has a flexible printed circuit board (FPC) on the top, z-axis anisotropic conductive tape in the middle and the PVDF film at the bottom. The PVDF film has a one-piece ground electrode at its bottom surface. Each sensor has 16 independent channels formed by the FPC. The center-to-center spacing of the receiving elements is 2 mm. The width of the element is 1.2 mm, which is narrow enough to produce uniform voltage response versus wavelength, but also wide enough to provide good electrical connection to the FPC through the conductive tape. The PVDF film is 110 µm thick and the total footprint of the PVDF sensor is 50 mm × 50 mm. The overall sensor thickness of 300 µm allows it to be very flexible. There are thinner PVDF films available that will provide better flexibility. However, since the PVDF film is very thin and will deform with the plate surface, the same amount of strain will be induced to the PVDF film regardless of how thick it is. Thus, the electric field is independent of film thickness, but thick films will have higher measured voltages. The 110 µm thick film is selected as a compromise between signal-to-noise ratio and flexibility.

## 5. Experiments

Two multi-element PVDF array sensors with 0° and 45° oriented PVDF films are bonded to a 1 mm-thick aluminum plate with cyanoacrylate. Three experiments are performed to test the performance of the sensor. The first two experiments provide a comparison of the sensor capability to receive Lamb and SH waves. The sensitivity will be compared with the numerical simulations. The third experiment uses the 45° oriented PVDF film sensor to receive Lamb and SH waves that arrive at the sensor simultaneously. Its capability to resolve two incident waves in the wavenumber-frequency domain will be demonstrated.

### 5.1. Sensitivity to Lamb Waves

**Figure 8:**
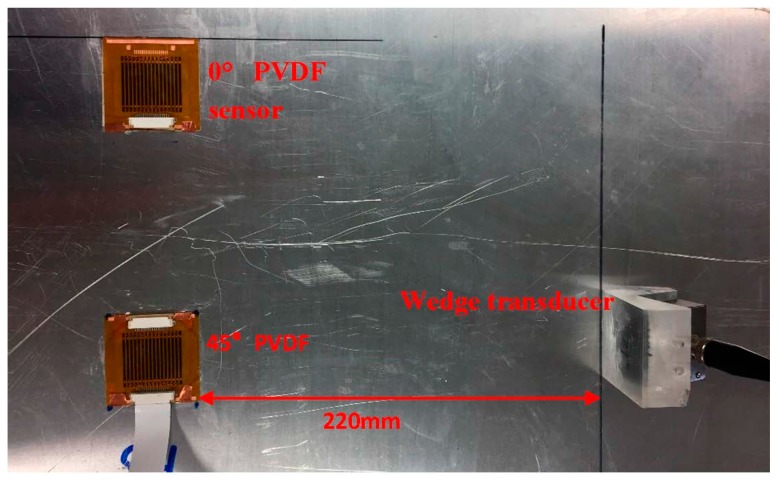
Experimental setup for PVDF sensors to receive Lamb waves.

The experimental setup is shown in Figure 8. A broadband 0.5 MHz angle wedge transducer oriented at 21° is used to excite Lamb waves. The excitation voltage is 100 V and the signal is a 5-cycle toneburst. Both A0 and S0 modes are excited because this incident angle does not match the phase velocity of either mode and the incident wave energy is distributed to both modes. Water is used as the couplant to maintain a uniform coupling condition when moving the transmitter. The excitation frequency is swept from 0.2 to 0.7 MHz.

**Figure 9:**
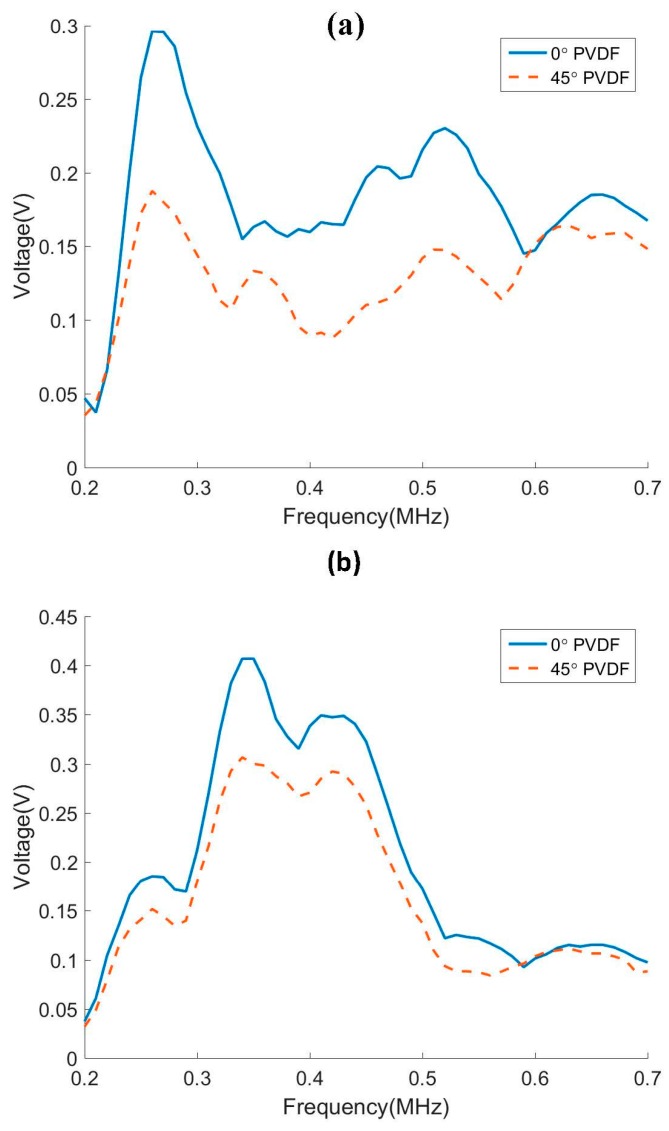
The sensitivity of 0° and 45° oriented PVDF sensors to the incident (**a**) A0 mode and (**b**) S0 mode.

The A0 and S0 modes can be resolved by the sensor because the multi-element sensor can extract the wave amplitude of a single mode by performing a 2D Fourier transform on the received signals [23]. The peak amplitudes of the A0 and S0 modes are plotted in Figure 9a,b respectively. The experimental results agree well with the numerical simulations in that the 0° PVDF film has better sensitivity to Lamb wave than the 45° PVDF film. This is confirmed for both A0 and S0 incidence.

### 5.2. Sensitivity to Shear Horizontal Waves

**Figure 10:**
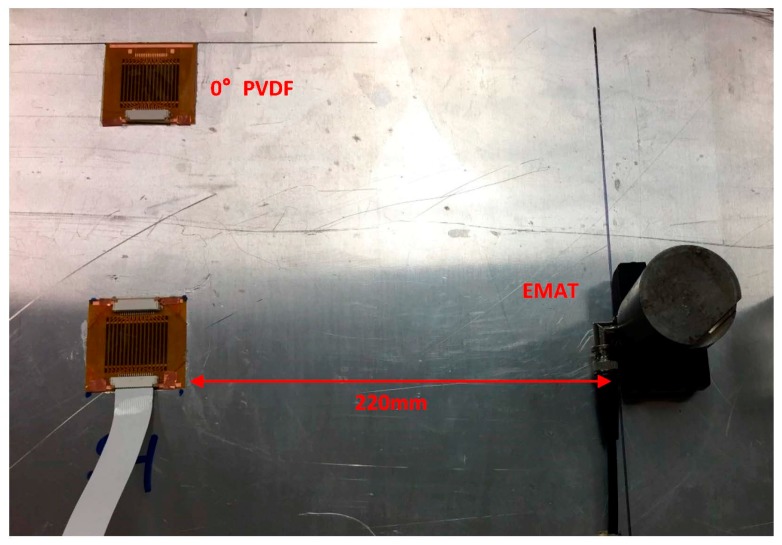
Experimental setup for PVDF sensors to receive SH waves.

The second experimental setup is similar to the first one except that the transmitter is an electro-magnetic acoustic transducer (EMAT) to excite SH waves, as shown in Figure 10. The EMAT has a fixed excitation wavelength and only the SH0 mode around 250 kHz can be excited effectively. Thus, the excitation frequency is swept only from 0.2 to 0.3 MHz. Similarly, the excitation voltage is 100 V and a 5-cycle toneburst is used as the excitation signal.

**Figure 11:**
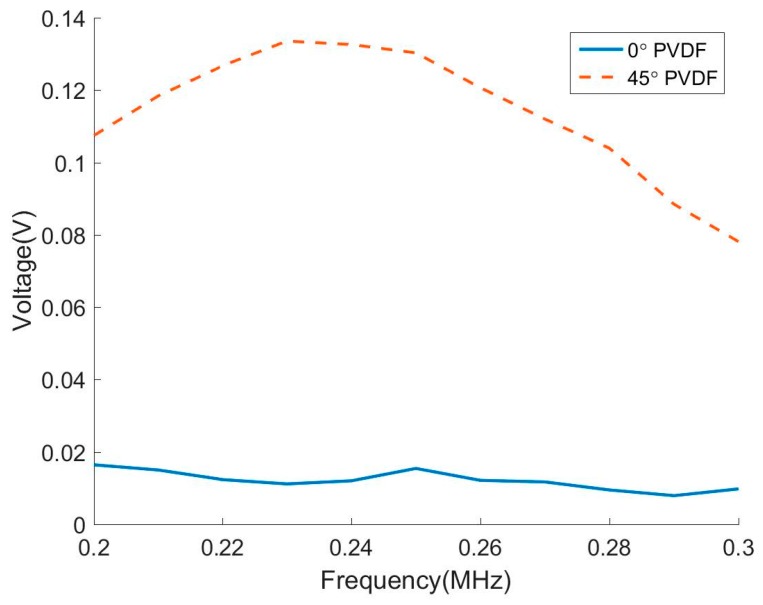
The sensitivity of 0° and 45° oriented PVDF sensors to the incident SH0 mode.

The experimental results for the peak amplitude are shown in Figure 11 from 0° and 45° oriented PVDF films for SH0 mode incidence. The sensitivity of 0° PVDF film to the SH0 mode is practically zero and is much weaker than the 45° PVDF film. This confirms the predictions and the capability of 45° PVDF film to receive SH waves.

### 5.3. Multi-Mode Reception and Decomposition

This experiment demonstrates the modal decomposition of multi-modal mixed signals. Both angle wedge transducer and EMAT are used to send guided waves to the 45° PVDF array sensor simultaneously. Since the EMAT can only excite the SH0 wave at 0.25 MHz, both transmitters are set to excite signals at 0.25 MHz.

**Figure 12:**
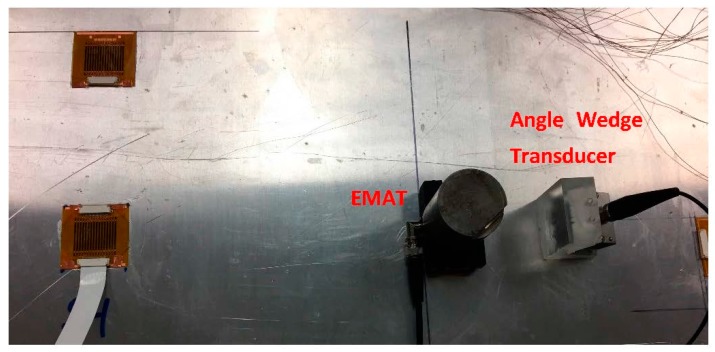
Experimental setup for modal decomposition. An angle wedge transducer and EMAT are used to generate mixed wave simultaneously.

**Figure 13:**
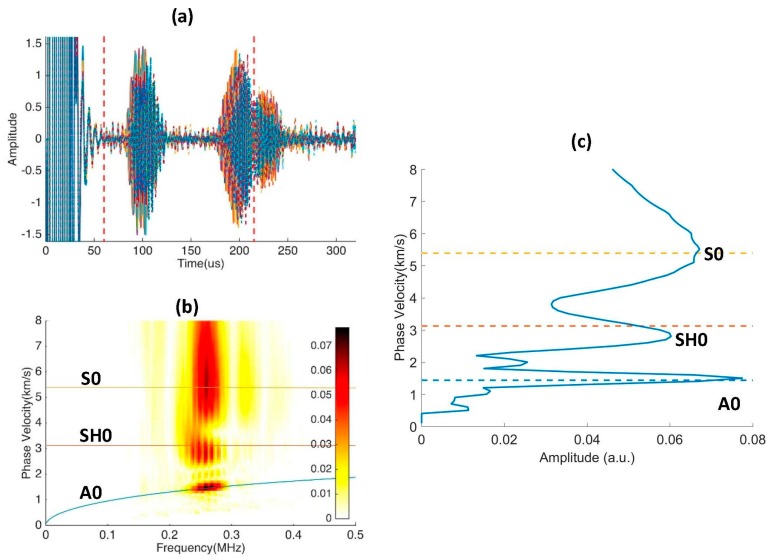
Experiment results for modal decomposition experiment. Signal window is taken from 60 to 220 µs to include all incident modes. (**a**) A-scan signals of 16 channels in the multi-element PVDF sensor; (**b**) Phase velocity-frequency spectrum superimposed on phase velocity dispersion curves; (**c**) Phase velocity spectrum showing the amplitude of the A0, SH0, and S0 modes.

The propagation distances from the two transmitters to the sensor are adjusted such that S0 and SH0 waves arrive at the sensor at the same time, as shown in Figure 12. The multi-element PVDF sensor has 16 channels and the corresponding A-scan signals are shown in Figure 13a. There are two wave packages between 60 and 220 µs, and the signals after 220 µs are back wall reflections and thus are not included in the analysis. The A-scan signals at this interval are selected for 2D Fast Fourier Transformation (2DFFT). The wavenumber-frequency spectrum is produced by conducting 2DFFT and then converted to the phase velocity-frequency spectrum and superimposed on the predicted dispersion curves in Figure 13b [23,24]. Clearly, all three fundamental modes are present. Figure 13c shows that the modal amplitudes of the three modes have comparable magnitudes [24].

**Figure 14:**
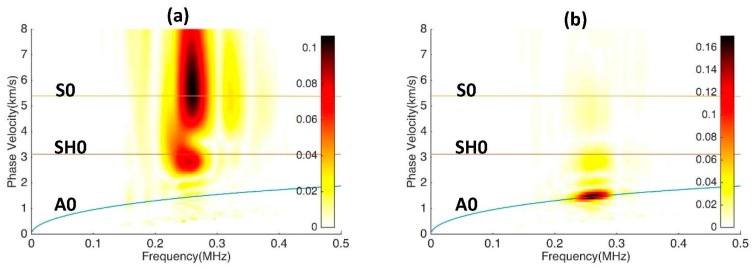
Experimental results for modal decomposition experiment. Signal windows are taken at (**a**) 60 to 150 µs and (**b**) 150 to 220 µs.

To further examine the signal of mixed wave modes, the signals received between 60–150 µs and then 150–220 µs are analyzed separately and the results are shown in Figure 14. Figure 14a indicates that the wave package arriving between 60–150 µs contains both S0 and SH0 modes. While both modes have the same arrival time (by design), they have completely different polarizations and would be difficult to resolve with other types of receivers. Figure 14b shows that the second wave package is the A0 mode, which has a slower group velocity. If desired, each wave mode can be filtered out by performing an inverse 2D Fourier transform and thus producing A-scan signals containing only one mode.

## 6. Conclusions

This work demonstrates the use of 45°-oriented PVDF film in multi-element array sensors. These novel sensors can detect both Lamb and SH waves, which have different polarizations, and resolve the received modes into independent modal amplitudes. The cost for the capability to receive both Lamb and SH waves is a lower sensitivity to Lamb waves relative to a 0° PVDF orientation. Furthermore, because of the multiple elements, the sensor is able to resolve different modes and produce relatively uniform voltage response to various wavelengths due to the small electrode size.

The PVDF sensor detects all three possible wave polarizations at the surface of a plate structure, which means that it is sensitive to any type of guided wave mode as long as there is displacement at the plate surface. Thus, the detectable waves are not limited to Lamb and SH waves in plate structures, and surface waves like Rayleigh and Love waves in half spaces as well as longitudinal and torsional waves in cylinder structures can also be detected. An inspection using one sensor to receive different guided wave modes could maximize the probability of detecting defects with different shapes and orientations; in some cases it provides valuable redundancy without having additional sensors. Thus, this sensor is a versatile tool in both the research laboratory environment and real SHM practice.

## Figures and Tables

**Table 1:** Material properties of PVDF film.

Symbol	Description	Value	Units
ρ	Mass density	1780	$\mathrm{kg}/m^{3}$
Y	Young’s modulus	3	GPa
ν	Poisson’s ratio	0.4	-
$d_{31}$	Piezo Strain constants	23	$10^{-12}\;m/V$
$d_{32}$		2	
$d_{33}$		−33	
ε	Permittivity	106	$10^{-12\;}F/m$
